# VELYS robotic-assisted total knee replacement leads to improved mobility, reduction in hospitalisation, surgical duration, and better psychological outcomes: a propensity score matched analysis

**DOI:** 10.1186/s42836-025-00342-x

**Published:** 2025-11-03

**Authors:** Jiawei Chen, Hong Yu Jared Chua, Jeremy Tze En Lim, Darren Keng-Jin Tay, Mann Hong Tan, Ming Han Lincoln Liow

**Affiliations:** 1 Yong Loo Lin School of Medicine, National University of Singapore, Singapore, Singapore; 2 Department of Orthopaedics, Singapore General Hospital, Singapore Health Services, Singapore, Singapore

**Keywords:** VELYS, ATTUNE, Robotic-assisted, TKA, Functional outcomes, Early outcomes

## Abstract

**Background:**

Robotic TKA (rTKA) was developed to improve implant positioning and accuracy of bone cuts, potentially resulting in improved functional outcomes for the patient. The Depuy Synthes VELYS™ Robotic-Assisted Solution (VRAS) is one of the latest, imageless systems available and utilizes the ATTUNE™ primary knee system. Due to its recency, there is limited literature on the outcomes of VRAS compared to its conventional total knee arthroplasty (cTKA) counterpart. This paper aims to look at the differences between VRAS and cTKA regarding early postoperative and 6-month functional outcomes.

**Methods:**

Registry data of all primary TKAs performed by 3 surgeons from January 2021 to December 2024 from a high-volume arthroplasty center were analysed. Patients who underwent VRAS or cTKA using ATTUNE™ implants were included. Propensity scores were estimated using logistic regression, followed by optimal matching in a 1:1 ratio to establish the VRAS and cTKA groups. Early postoperative outcomes (static/dynamic pain score, ambulation distance, length of stay), 6-month functional outcomes (Knee Society Score, Oxford Knee Score, SF-36, patient expectation/satisfaction scores), and proportion attaining a minimum clinically important difference (MCID) were analysed.

**Results:**

Sixty-five VRAS patients were matched with 65 in the cTKA group. The VRAS showed significantly shorter surgical duration (78.2 vs. 85.5 min, *P* = 0.04), improved ambulation distance (22.2 vs. 11.3 m, *P* < 0.001), and shorter length of stay (2.48 vs. 3.66 days, *P* = 0.01). Both groups showed significant improvements in the majority of the functional outcome scores at 6 months. The VRAS displayed a trend towards higher SF-36 outcome measures, with significant differences in SF-36 vitality (*P* = 0.001), SF-36 mental component summary (*P* = 0.015), and a larger proportion of patients achieving SF-36 bodily pain MCID (76.9 vs 60.0%, *P* = 0.038). More patients reported satisfaction and expectation fulfillment (95.2% vs 92.3% and 92.1% vs 87.7%, respectively), albeit non-significantly (*P* = 0.718 and *P* = 0.413).

**Conclusion:**

The VRAS TKA demonstrated superior immediate postoperative advantages and patient-reported functional outcomes at 6 months. Further studies are needed to determine long-term outcomes.

Trial registration.

Centralized Institutional Review Board (CIRB: 2024–4046).

**Supplementary Information:**

The online version contains supplementary material available at 10.1186/s42836-025-00342-x.

## Introduction

Total knee arthroplasty (TKA) is the gold standard treatment for symptomatic end-stage knee osteoarthritis (OA) involving multiple compartments of the knee [1]. Robotic TKA (rTKA) was developed to improve implant positioning and accuracy of bone cuts, potentially resulting in improved functional outcomes for the patient [2, 3]. The DePuy Synthes VELYS™ Robotic-Assisted Solution (VRAS) (DePuy Synthes, Johnson & Johnson MedTech, Warsaw, NJ, USA) is a table-mounted system that utilizes imageless intra-operative registration to guide ligament balancing and saw cuts [4].

There exists a variety of rTKA systems, both image-based and imageless systems, with outcomes that have been extensively studied compared to their conventional TKA (cTKA) counterparts. One such CT image-based system is the MAKO® robotic-assisted TKA, which has been the most extensively reported robotic platform in the English-language orthopaedic literature. A systematic review of 26 studies by Batailler et al. (2021) showed that use of the MAKO® system improved implant positioning, significantly reduced postoperative pain and time to discharge, and resulted in modest improvements in certain functional outcomes at one year compared to cTKA [5]. A systematic review and meta-analysis of 17 studies by Zaidi et al. (2024) looked at the ROSA® imageless TKA system and found improved accuracy and functional scores, with no difference in complication rates compared to cTKA [6].

There is limited literature regarding the outcomes of the VRAS system when compared to its cTKA, which utilizes ATTUNE™ implants. Current literature highlights VRAS’s accuracy in femoral and tibial component positioning and reducing unintended outliers compared to cTKA in both cadaveric and clinical studies [4, 7, 8]. Huang et al. (2024) also showed lower revisit and knee-related readmission rates post VRAS compared to cTKA. The authors also reported no significant cost differences between groups, citing decreased length of stay (LOS), fewer opioid prescriptions, and reduced resources utilized [9]. Whether VRAS’s improvements in accuracy and positioning translate to enhanced postoperative functional outcomes and patient satisfaction is not well studied. This paper aimed to study the differences between VRAS and cTKA, examining early postoperative, 6-month functional outcomes, and patient satisfaction.

## Materials and methods

### Study design

We conducted a retrospective, propensity score-matched cohort study of 154 patients who underwent primary unilateral TKA for uncomplicated end-stage knee OA using ATTUNE implants from January 2022 to December 2024, before and after the introduction of VRAS at the institution. The main inclusion and exclusion criteria are listed in Table 1. The Centralized Institutional Review Board and Ethics Committee’s approval was sought before the commencement of the project (CIRB: 2024–4046). Patient data was extracted from the institution’s TKA registry and anonymized for analysis.

**Table 1:** Inclusion and Exclusion Criteria

Inclusion criteria	Exclusion criteria
Patients undergoing Primary TKA	Patients undergoing revision TKA
Patients undergoing unilateral TKA	Patients undergoing bilateral TKA
Patients undergoing TKA for primary end-stage knee OA	Patients undergoing TKA for indications other than primary knee OA (e.g., inflammatory arthritis)
Use of either the VELYS robotic system with ATTUNE implant or a conventional ATTUNE implant	Use of robotic systems other than VELYS, or use of non-ATTUNE implants
Documentation of early outcome data (e.g., Surgical Duration, post-operative pain scores, LOS, and ambulation distance) and pre-operative and 6 months functional outcomes (e.g., KSS, SF-36)	Missing early outcome data, pre-operative or 6 months functional data (e.g., from lost-to-follow-up)

TKA: total knee replacement; OA: osteoarthritis; LOS: length of stay; KSS: knee society score; SF-36: short-form 36

A total of 89 patients underwent robotic-assisted surgery (VELYS, Depuy Synthes, ATTUNE CR) and 65 patients underwent conventional TKA (Depuy Synthes, ATTUNE CR). These were the first such VRAS cases performed in our institution with available 6-month follow-up data for analysis, and the surgeons had no prior experience with VRAS in other settings before its introduction in our institution. After performing propensity score matching, 65 patients were available in each group. A post-hoc power analysis based on ambulation distance and SF-36 vitality confirmed that this sample size was adequate to detect meaningful differences between groups (see *Statistical analysis*). In the VRAS group, 71% of cases were performed by surgeon A, 22% by surgeon B, and 7% by surgeon C. In the cTKA group, 48% of cases were performed by surgeon A, 48% by surgeon B, and 5% by surgeon C. Patients’ demographic parameters, such as age, race, height, body mass index (BMI), and male proportion, were recorded.

### Surgical technique

All surgeries were performed by one of three experienced arthroplasty surgeons in a single high-volume center for both robotic and conventional groups. The VELYS Robotic-Assisted Solution with software version 1.8 was used in all robotic-assisted procedures and completed without robot malfunction or the need for conversion to the manual technique. All patients received the DePuy Synthes ATTUNE Knee system implants. All surgeons utilised a cruciate retaining (CR) implant with a cemented fixation method across both VRAS and cTKA groups.

**Fig. 1:**
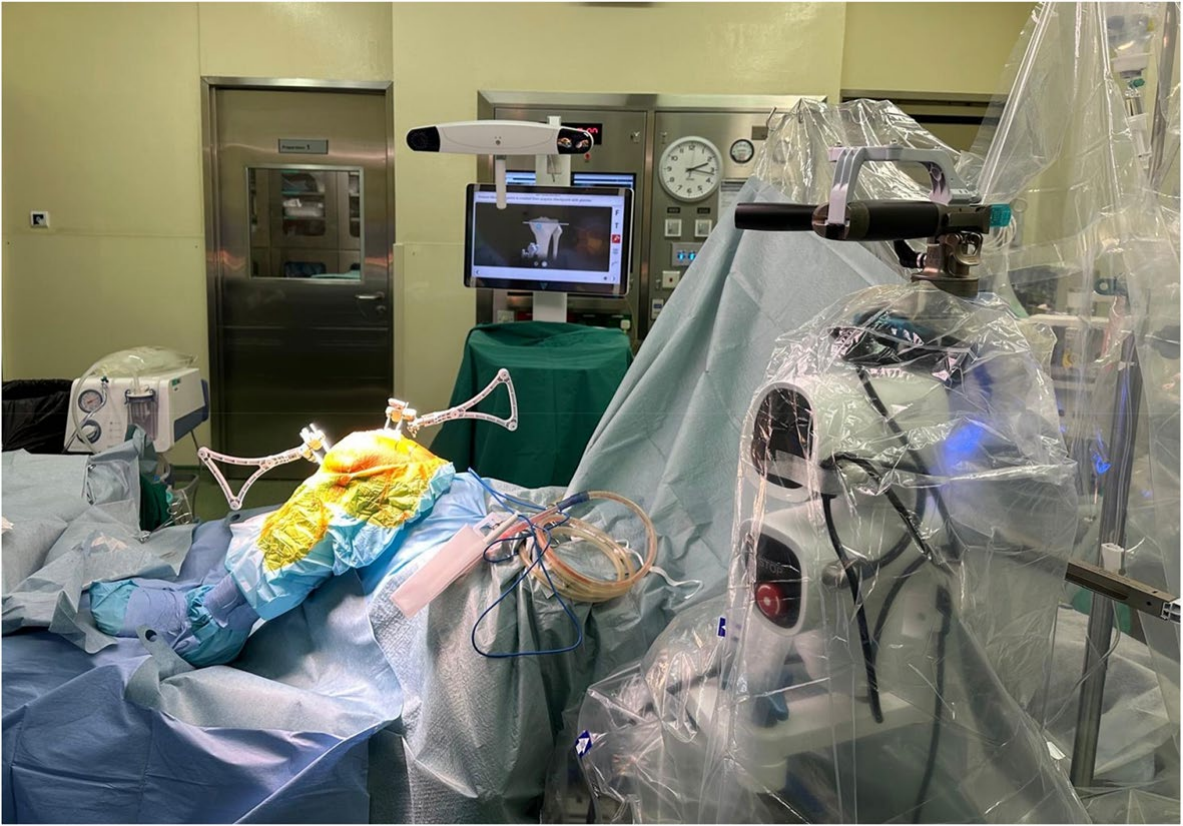
Image of the VRAS imageless, table-mounted set-up with femoral and tibial arrays in situ

**Fig. 2:**
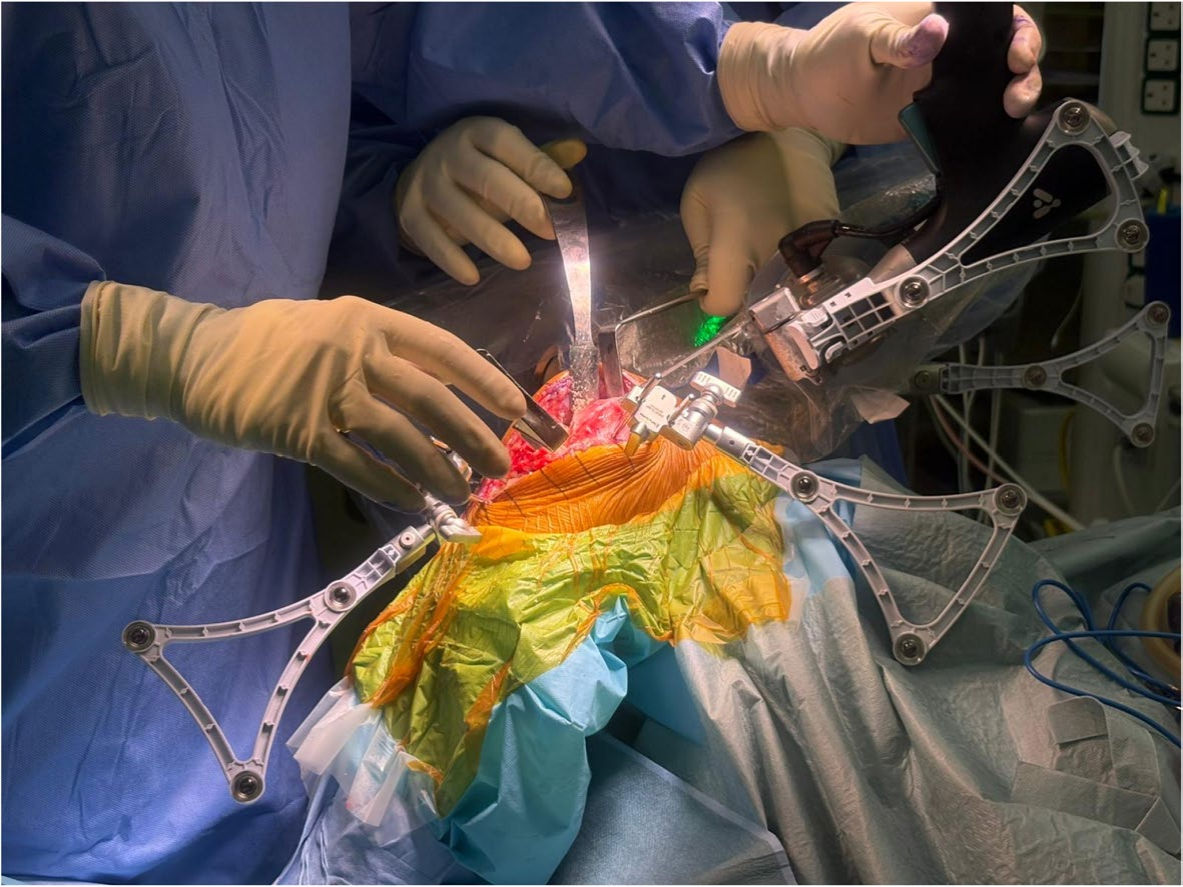
Image of the oscillating saw being positioned in its precise saw plane

Spinal anaesthesia was performed by our institution’s anaesthetist pre-operatively. A standard medial parapatellar approach was performed for all patients. In the VRAS group, registration pins and arrays were placed intra-incisionally in the medial femoral condyle and extra-incisionally in the medial proximal tibia, followed by a registration process that identified anatomical landmarks of the knee and the center of the hip and ankle (Fig. 1**)**. The patient’s initial lower limb alignment and correctability were determined by assessing the soft tissue envelope through the full knee range of motion and varus/valgus stress poses. An initial surgical plan was generated by the system, which was further adjusted by the surgeon to achieve ideal gap balancing. After planning, the system positioned the oscillating saw to the precise saw plane, allowing the surgeon to perform the necessary bony cuts **(**Fig. 2**)**. Trial implants were inserted to ensure proper balancing and alignment before the actual implants were implanted. All procedures were performed following the manufacturer’s recommended standard surgical technique. In the cTKA group, a standardized technique to achieve mechanical alignment was used, which adhered to the manufacturer’s instrumentation instructions using the provided proprietary jigs.

For all patients, patelloplasty (including lateral facetectomy and removal of patella osteophytes) was performed. Patellar resurfacing was performed only by surgeon B. Before closure of the capsule, dilute betadine irrigation was performed, followed by administration of local periarticular analgesia. In addition, Surgeon A also administered an adductor canal block. Tranexamic acid was applied topically before the capsule and skin were closed. All patients received postoperative physiotherapy and standard wound care according to institutional guidelines.

### Clinical evaluation

On postoperative day 1, inpatient physiotherapists instructed patients to perform standardized exercises. Static and dynamic pain scores (using the Visual Analog Scale) and ambulation distance were assessed during this time. Together with surgical duration and LOS, these parameters form the early postoperative outcomes in this study.

Patients were reviewed in an outpatient setting by experienced physiotherapists pre-operatively and at 6 months postoperatively in our institution. During each visit, a range of motion (flexion and extension), Oxford Knee Score (OKS), Knee Society knee and function scores (KSS), and Short-Form 36 (SF36) health survey questionnaire were administered.

The OKS comprises 12 questions that evaluate pain and function of the patient. Each question is rated from 1 to 5, from best to worst outcome. The scores of all questions were added, and the overall score ranges from 12 (best) to 60 (worst) [10].

The KSS knee and function scores assess objective (pain, knee alignment, ROM) and subjective (function, e.g., stairs, walking, use of walking aid) outcomes [11]. Each of the knee and function scores is individually rated on a scale of 0 to 100, from worst to best outcome, respectively.

The SF36 health survey measures 8 domains of physical function (PF), role physical (RP), bodily pain (BP), general health (GH), vitality (VI), social function (SF), role emotional (RE), and mental health (MH). Scores for each domain range from 0 to 100, with a higher score indicating better outcomes. These domains can be further categorized into the physical component summary (PCS) and mental component summary (MCS) [12].

The proportion of patients attaining minimum clinically important difference (MCID) in both VRAS and cTKA groups was calculated. The following 6-month MCID thresholds were taken from current literature: OKS (5 points), KSS function score (10 points), KSS knee score (9 points), SF36-PF (11.6 points), SF36-RP (11.7 points), SF36-BP (16.9 points), SF36-GH (0.85 points), SF36-VI (3.9 points), SF36-SF (11.7 points), SF36-RE (7.7 points), SF36-MH (− 0.3 points), SF36 PCS (10 points) and MCS (10 points) [13–16].

Additionally, at 6 months, patient satisfaction and fulfilment of expectations are recorded. Satisfaction score was rated from 1 to 7, while fulfilment of expectations was rated from 1 to 6. A score of 1 to 3 for both scores indicates patient satisfaction and expectations fulfilled, while a score of more than 3 indicates dissatisfaction and expectations not being fulfilled.

### Statistical analysis

Statistical analysis was conducted using RStudio (Version 2022.12.0 + 353, Posit, PBC, Boston, MA, USA). A post-hoc power analysis was conducted using ambulation distance and SF-36 vitality as key outcomes using a power of 0.9 and significance of 0.05. The required sample size was 38 and 63 for ambulation distance and SF-36 vitality, respectively. Given a sample size of 65 per group, this study is adequately powered at 90% to detect meaningful differences in outcome measures.

Propensity score matching was performed to reduce confounding bias. Propensity scores were estimated using logistic regression, based on demographic variables including age, gender, and BMI. This was followed by optimal matching in a 1:1 ratio to establish the VRAS and cTKA groups. A total of 65 VRAS patients were matched with 65 cTKA patients successfully.

Continuous data were presented as mean and standard deviation. Statistical significance was defined as *P* ≤ 0.05, which is the convention for type I errors. Student’s unpaired t-test was used to compare quantitative variables between both groups. The mean difference between groups and 95% confidence interval (CI) were recorded. A chi-squared test for proportions was used to compare categorical variables (e.g., satisfaction/expectation, proportion of patients meeting MCID).

## Results

### Demographics

A total of 65 patients in the VRAS group were matched with 65 patients in the cTKA group. There were no significant differences in gender proportions (*P* = 1), mean BMI (*P* = 0.728), age of the patients (*P* = 0.840), and ASA distribution (*P* = 0.736) between groups (Table 2). All cases in both groups received CR implants with cemented fixation. Patellar resurfacing was performed in 22% of the VRAS group and 48% of the cTKA group. The distribution of propensity scores for unmatched and matched cohorts is available as a histogram and jitter plot in the supplementary material.

**Table 2:** Baseline patient demographics

	**Robotic-assisted**	**Conventional**	**Significance/*P*-value**
Total No. of knees (patients)	65	65	
Gender			
Male	16	16	1 (n.s.)
Female	49	49	
BMI/kg·m^−2^	28.1 ± 4.59	27.8 ± 4.24	0.728 (n.s.)
Age/years	70.5 ± 8.58	70.2 ± 8.59	0.840 (n.s.)
ASA	I: 3 II: 52 III: 10	I: 3 II: 55 III: 7	0.736 (n.s.)

BMI and age are reported as mean ± standard deviation

BMI: body mass index, n.s.: not significant

Both VRAS and cTKA groups showed significant improvements in the majority of the functional outcome scores at 6 months compared to pre-operatively (*P* < 0.001 to 0.024). However, the cTKA group had a higher number of functional outcomes, which did not demonstrate significant differences (SF36 general health, vitality, role emotional, mental health, and MCS) (Table 3).

**Table 3:** Functional outcomes at pre-op and 6 months for the robotic-assisted (*n* = 65) and conventional (*n* = 65) TKA groups

Functional Outcomes	Robotic Assisted			Conventional		
	**Pre-op**	**6 months**	**P-value**	**Pre-op**	**6 months**	***P*-value**
Range of motion						
Extension	4.43 ± 6.02	2.63 ± 5.39	0.024	5.61 ± 5.27	2.08 ± 3.69	(< 0.001)
Flexion	114.9 ± 21.2	110.4 ± 15.2	0.018	117.4 ± 17.9	112.5 ± 13.7	0.016
Oxford Knee Score (OKS)*	36.2 ± 9.07	21.8 ± 8.01	(< 0.001)	35.2 ± 7.23	21.7 ± 7.33	(< 0.001)
KSS Function score	46.2 ± 24.4	63.8 ± 27.9	(< 0.001)	48.8 ± 20.2	61.8 ± 26.4	(< 0.001)
KSS Knee score	37.2 ± 14.0	82.9 ± 15.6	(< 0.001)	38.6 ± 16.9	82.3 ± 15.5	(< 0.001)
SF-36 physical function	36.7 ± 26.5	59.9 ± 30.4	(< 0.001)	38.5 ± 22.6	60.2 ± 31.0	(< 0.001)
SF-36 role physical	17.3 ± 35.9	56.9 ± 45.2	(< 0.001)	11.9 ± 30.0	52.7 ± 45.3	(< 0.001)
SF-36 bodily pain	31.2 ± 19.4	65.7 ± 25.8	(< 0.001)	33.2 ± 18.1	60.6 ± 29.5	(< 0.001)
SF-36 general health	69.1 ± 17.8	75.0 ± 14.8	0.009	68.3 ± 20.4	73.0 ± 17.2	0.096 (n.s.)
SF-36 vitality	73.8 ± 19.6	81.4 ± 14.5	0.002	71.4 ± 20.6	71.4 ± 20.0	1 (n.s.)
SF-36 social function	56.0 ± 38.9	83.3 ± 31.3	(< 0.001)	55.6 ± 32.9	80.8 ± 31.7	(< 0.001)
SF-36 role emotional	96.9 ± 17.4	96.9 ± 17.4	1 (n.s.)	97.9 ± 13.0	96.9 ± 15.3	0.686 (n.s.)
SF-36 mental health	82.6 ± 16.3	87.3 ± 14.5	0.011	83.0 ± 14.1	83.1 ± 14.4	0.934 (n.s.)
SF-36 PCS	32.2 ± 9.51	43.3 ± 11.5	(< 0.001)	32.1 ± 7.44	43.6 ± 11.4	(< 0.001)
SF-36 MCS	53.4 ± 10.6	58.8 ± 8.61	(< 0.001)	53.1 ± 11.1	54.9 ± 9.51	0.227 (n.s.)

^*^ OKS: lower scores indicate better outcomes

KSS: knee society score; SF-36: short-form 36; PCS: physical component summary; MCS: mental component summary

### Early postoperative outcomes

The VRAS group showed a significantly shorter surgical duration (78.2 ± 21.6 vs 85.5 ± 19.2, *P* = 0.044), improved POD1 ambulation distance (22.2 ± 18.4 vs 11.3 ± 8.72, *P* < 0.001), and decreased time to discharge (2.48 ± 2.17 vs 3.66 ± 2.97, *P* = 0.011). The VRAS also had a lower POD1 static pain score (1.2 ± 1.66 vs 1.77 ± 2.15); however, this was not statistically significant (*P* = 0.094). No difference in POD1 dynamic pain scores was noted between both VRAS and cTKA groups (*P* = 0.970) (Table 4).

**Table 4:** Comparison of early post-operative outcomes between robotic-assisted and conventional TKA groups (surgical duration, static and dynamic pain scores, ambulation distance, and time to discharge)

	**Robotic-assisted**		**Conventional**		**Mean difference**	**95% CI of mean difference**	***P*-value**
	**Mean ± SD**	**95% CI**	**Mean ± SD**	**95% CI**			
Surgical duration/min	78.2 ± 21.6	5.34	85.5 ± 19.2	4.77	7.3	0.213 to 14.4	0.0436
Pain score (VAS)							
Static	1.2 ± 1.66	0.411	1.77 ± 2.15	0.532	0.57	− 0.098 to 1.24	0.094 (n.s.)
Dynamic	4.22 ± 2.22	0.550	4.20 ± 2.48	0.615	0.02	* − *0.833 to 0.802	0.970 (n.s.)
Ambulation distance/m	22.2 ± 18.4	4.57	11.3 ± 8.72	2.16	10.9	* − *15.9 to − 5.84	< 0.001
Time to Discharge/days	2.48 ± 2.17	0.537	3.66 ± 2.97	0735	1.18	0.279 to 2.08	0.011

SD: standard deviation; CI: confidence interval; VAS: visual analog scale

### Six-month functional outcomes

No significant differences were observed between groups in terms of range of motion (flexion and extension), OKS, KSS function, and knee scores at 6 months (Table 5). The VRAS group displayed a trend towards higher SF-36 outcome measures, with significant differences in SF-36 vitality (81.4 ± 14.5 vs 71.4 ± 20.0, *P* = 0.001) and SF-36 mental component summary (58.8 ± 8.61 vs 54.9 ± 9.51, *P* = 0.015) (Table 6).

**Table 5:** Comparison of pre-operative and 6 months functional outcomes between robotic-assisted and conventional TKA groups (ROM, OKS, KSS)

	**Robotic-assisted**		**Conventional**		**Mean difference**	**95% CI of mean difference**	***P*-value**
	**Mean ± SD**	**95% CI**	**Mean ± SD**	**95% CI**			
*Range of Motion*							
*Extension*							
Pre-op	4.43 ± 6.02	1.49	5.61 ± 5.27	1.32	1.18	* − *0.792 to 3.15	0.239 (n.s.)
6 months	2.63 ± 5.39	1.34	2.08 ± 3.69	0.953	0.55	* − *2.17 to 1.08	0.506 (n.s.)
*Flexion*							
Pre-op	114.9 ± 21.2	5.25	117.4 ± 17.9	4.48	2.5	* − *4.28 to 9.40	0.460 (n.s.)
6 months	110.4 ± 15.2	3.77	112.5 ± 13.7	3.53	2.1	* − *3.04 to 7.18	0.425 (n.s.)
Oxford Knee Score (OKS)*							
Pre-op	36.2 ± 9.07	2.25	35.2 ± 7.23	1.79	1.0	* − *3.85 to 1.85	0.488 (n.s.)
6 months	21.8 ± 8.01	1.98	21.7 ± 7.33	1.82	0.1	* − *2.74 to 2.59	0.955 (n.s.)
*KSS Function score*							
Pre-op	46.2 ± 24.4	6.06	48.8 ± 20.2	5.02	2.6	* − *5.18 to 10.4	0.508 (n.s.)
6 months	63.8 ± 27.9	6.90	61.8 ± 26.4	6.54	2.0	* − *11.4 to 7.42	0.675 (n.s.)
*KSS knee score*							
Pre-op	37.2 ± 14.0	3.47	38.6 ± 16.9	4.21	1.4	* − *4.03 to 6.78	0.615 (n.s.)
6 months	82.9 ± 15.6	3.87	82.3 ± 15.5	4.01	0.6	* − *6.08 to 4.96	0.841 (n.s.)

^*^ OKS: lower scores indicate better outcomes

SD: standard deviation; CI: confidence interval; KSS: knee society score

**Table 6:** Comparison of pre-operative and 6 months functional outcomes between robotic-assisted and conventional TKA groups (SF-36)

	**Robotic-assisted**		**Conventional**		**Mean difference**	**95% CI of mean difference**	***P*-value**
	**Mean ± SD**	**95% CI**	**Mean ± SD**	**95% CI**			
*SF-36 physical function*							
Pre-op	36.7 ± 26.5	6.56	38.5 ± 22.6	5.60	1.8	* − *6.78 to 10.3	0.683 (n.s.)
6 months	59.9 ± 30.4	7.53	60.2 ± 31.0	7.68	0.3	* − *10.3 to 11.0	0.955 (n.s.)
*SF-36 role physical*							
Pre-op	17.3 ± 35.9	8.89	11.9 ± 30.0	7.44	5.4	* − *16.9 to 6.10	0.355 (n.s.)
6 months	56.9 ± 45.2	11.2	52.7 ± 45.3	11.2	4.2	* − *19.9 to 11.5	0.595 (n.s.)
*SF-36 bodily pain*							
Pre-op	31.2 ± 19.4	4.81	33.2 ± 18.1	4.49	2.0	* − *4.55 to 8.49	0.551 (n.s.)
v6 months	65.7 ± 25.8	6.39	60.6 ± 29.5	7.31	5.1	* − *14.8 to 4.47	0.291 (n.s.)
*SF-36 general health*							
Pre-op	69.1 ± 17.8	4.42	68.3 ± 20.4	5.05	0.8	* − *7.45 to 5.85	0.812 (n.s.)
6 months	75.0 ± 14.8	3.68	73.0 ± 17.2	4.27	2.0	* − *7.58 to 3.58	0.479 (n.s.)
*SF-36 vitality*							
Pre-op	73.8 ± 19.6	4.85	71.4 ± 20.6	5.10	2.4	* − *9.35 to 4.58	0.500 (n.s.)
6 months	81.4 ± 14.5	3.59	71.4 ± 20.0	4.96	10	* − *16.1 to − 3.93	0.001
*SF-36 social function*							
Pre-op	56.0 ± 38.9	9.64	55.6 ± 32.9	8.14	0.4	* − *12.9 to 12.1	0.952 (n.s.)
6 months	83.3 ± 31.3	7.77	80.8 ± 31.7	7.86	2.5	* − *13.4 to 8.45	0.652 (n.s.)
*SF-36 role emotional*							
Pre-op	96.9 ± 17.4	4.31	97.9 ± 13.0	3.22	1.0	* − *4.31 to 6.36	0.704 (n.s.)
6 months	96.9 ± 17.4	4.31	96.9 ± 15.3	3.79	0	* − *5.68 to 5.68	1 (n.s.)
*SF-36 mental health*							
Pre-op	82.6 ± 16.3	4.04	83.0 ± 14.1	3.49	0.4	* − *4.98 to 5.60	0.909 (n.s.)
6 months	87.3 ± 14.5	3.59	83.1 ± 14.4	3.57	4.2	* − *9.14 to 0.89	0.106 (n.s.)
*SF-36 PCS*							
Pre-op	32.2 ± 9.51	2.36	32.1 ± 7.44	1.84	0.1	* − *3.06 to 2.87	0.949 (n.s.)
6 months	43.3 ± 11.5	2.84	43.6 ± 11.4	2.82	0.3	* − *3.69 to 4.24	0.892 (n.s.)
*SF-36 MCS*							
Pre-op	53.4 ± 10.6	2.63	53.1 ± 11.1	2.74	0.3	* − *4.12 to 3.4	0.851 (n.s.)
6 months	58.8 ± 8.61	2.13	54.9 ± 9.51	2.36	3.9	* − *7.09 to − 0.792	0.015

SD: standard deviation; CI: confidence interval; SF-36: short-form 36; PCS: physical component summary; MCS: mental component summary

**Fig. 3:**
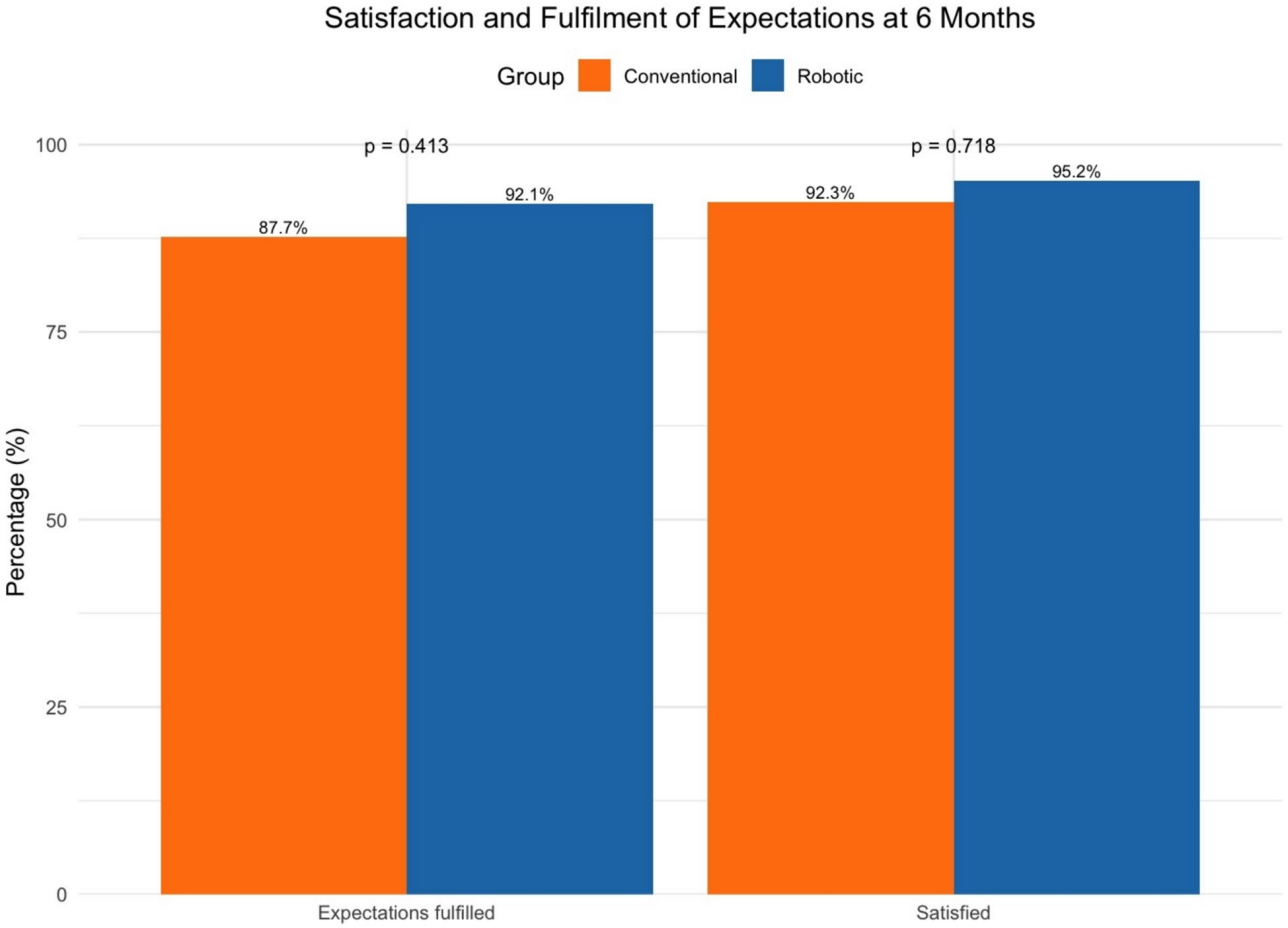
Bar graph of the percentage of satisfaction and expectations fulfilled at 6 months

Additionally, the VRAS group had greater proportions of patients achieving MCID in the majority of the domains compared to cTKA, although non-significant. There was a significantly larger proportion of patients achieving SF-36 bodily pain MCID (76.9 vs 60.0%, *P* = 0.038) in the VRAS group (Table 7). Furthermore, more patients reported 6-month satisfaction and expectation fulfilment (95.2 vs 92.3% and 92.1 vs 87.7% respectively) in the VRAS group; however, this was not statistically significant (*P* = 0.718 and *P* = 0.413) (Fig. 3).

**Table 7:** Comparison of the proportion achieving MCID at 6 months between robotic-assisted and conventional groups

Functional score	Robotic (%)	Conventional (%)	95% CI (%)	*P*-value
Oxford Knee Score (5)	83.1	86.2	* − *0.155 to 0.093	0.627 (n.s)
*Knee Society Score*				
Function Score (10)	72.3	56.9	* − *0.316 to 0.008	0.067 (n.s)
Knee Score (9)	96.9	91.5	* − *0.1370 to 0.029	0.193 (n.s)
*Short Form-36*				
Physical function (11.6)	56.9	58.5	* − *0.154 to 0.185	0.859 (n.s)
Role physical (11.7)	53.8	53.8	* − *0.171 to 0.171	1 (n.s)
Bodily pain (16.9)	76.9	60.0	* − *0.326 to − 0.012	0.038
General health (0.85)	55.4	53.8	* − *0.187 to 0.156	0.860 (n.s)
Vitality (3.9)	53.8	40	* − *0.308 to 0.031	0.114 (n.s)
Social function (11.7)	52.3	58.5	* − *0.109 to 0.232	0.480 (n.s)
Role emotional (7.7)	3.1	3.1	* − *0.059 to 0.059	1 (n.s)
Mental health (*− *0.3)	61.5	60.0	* − *0.183 to 0.152	0.857 (n.s)
PCS (10)	52.3	52.3	* − *0.172 to 0.172	1 (n.s)
MCS (10)	26.2	24.6	* − *0.165 to 0.134	0.840 (n.s)

PCS: physical component summary; MCS: mental component summary

## Discussion

The main findings of this study include superior early postoperative outcomes, including significantly shorter surgical duration, improved immediate post-operative ambulation distance, and shorter LOS. This is in line with several studies looking at VRAS and other rTKA systems, showing improvements in early postoperative outcomes [4, 17–20]. With regards to surgical duration, variability exists in the literature, with some studies reporting longer surgical durations in rTKA compared to cTKA, while others show no significant difference or even shorter durations [21, 22]. This is often multifactorial, including surgeon experience, progression on the learning curve, and complexity of the cases [19, 23, 24]. Being a high-volume arthroplasty centre, surgeons at our institution had experience with different rTKA systems with transferable skills, resulting in a reduced learning curve, which likely resulted in shorter surgical durations. Increased surgical duration has been associated with an increased risk of infection, with a critical operative duration of 127 min described for TKA [25]. Shorter surgical duration in rTKA is ideal and beneficial to reduce complication rates and the risk of requiring revision surgery due to infection.

Despite improvements in postoperative day 1 ambulation distance and decreased LOS, pain scores (static and dynamic) showed no significant differences between groups. In contrast, Marsawa et al. (2022), Kayani et al. (2018), and Bhimani et al. (2020) showed a significant reduction in pain scores postoperatively in various rTKA systems, with associated decreased LOS [18–20]. In our present study, a large proportion of patients actually report minimal to no pain on post-operative day 1. A total of 41.5% cTKA patients reported no static pain postoperatively, while a larger 56.9% VRAS patients reported no static pain. Similarly, 41.5% cTKA patients and 40% VRAS patients reported mild to no pain (0–2 on VAS) on movement. In various outcome measures, a floor or ceiling effect is considered present if proportions of patients at the extreme scores (highest or lowest) exceed 15% [26, 27]. These large proportions of patients exhibiting the lowest pain scores could contribute to a floor effect, thereby limiting any significant differences. It is of note that VRAS still portrays subtle improvements in post-operative static pain scores, and notable improvements in ambulation distance and LOS could be attributed to improvements in pain perceived by the patients.

While both VRAS and cTKA groups showed significant improvements in the majority of the functional outcome scores at 6 months compared to pre-operatively, VRAS displayed a trend towards higher SF-36 outcome measures, with significant differences in SF-36 vitality, mental component summary, and a larger proportion of patients achieving SF-36 bodily pain MCID. However, OKS and KSS knee and function scores showed no significant differences. Interestingly, the results of the current study are not unlike those found in a previous 2-year follow-up of a randomized controlled trial (RCT), looking at the use of the ROBODOC system in the same institution. Liow et al. (2016) found significant differences in SF36 outcomes scores (general health, vitality, and role emotional), and trends towards subtle improvements in functional scores in general, with no significant differences in OKS and KSS [28]. The concordance in outcome scores among different robotic systems suggests that rTKA improves patients’ perceived well-being and quality of life, beyond the benefits to knee alignment and implant positioning. Superior early postoperative outcomes in VRAS may be a contributory factor in improving patients’ psychological well-being and patient-reported energy levels, leading to notable benefits in SF-36 vitality and MCS scores. While superior functional advantages may not have translated into improvements in knee-specific scores like the OKS and KSS, this could indicate that while VRAS and cTKA both effectively restore knee function, VRAS provides additional benefits to patient-perceived recovery, owing to broader aspects of health-related quality of life outcomes measured by the SF36. These functional outcomes are also reflective of the eventual patient satisfaction and expectation fulfilment, whereby more patients reported satisfaction and expectation fulfilment in VRAS compared to cTKA, despite being non-significant.

Strengths of this study include: (1) propensity score matching to reduce effects of confounding, (2) use of the same ATTUNE implant across both groups, (3) surgeries performed at a single institution with similar postoperative protocols, (4) first few studies looking at early postoperative and functional outcomes of VRAS, (5) use of multiple validated knee outcome scores. However, this study is not without its limitations. Firstly, the retrospective nature of the study potentially introduces selection bias. This was mitigated by adjusting for confounding factors through propensity score matching, strengthening the validity of the comparison between groups. However, although propensity score matching was performed, this approach cannot control for unmeasured biases such as the Hawthorne effect. As our patients were not blinded to the treatment, their awareness of undergoing robotic surgery potentially led to the VRAS trending towards improved outcome measures. Secondly, due to the recency of VRAS, longer-term (e.g., 2 years) functional outcomes, satisfaction, and expectation fulfilment rates are not available, which may provide valuable insights. Thirdly, radiological outcomes were not included in this study. Hence, the relationship between intended implant positioning, alignment accuracy, and clinical outcomes could not be evaluated. Lastly, while the alignment philosophy of the VRAS group was largely functional, compared to a mechanical alignment approach in the cTKA group, the differences in outcomes may be partially attributable to differences in alignment strategy rather than robotic assistance alone. However, multiple recent RCTs do suggest that a difference in alignment strategy may not have a significant impact on patients’ early and functional outcomes [29–31]. Nevertheless, it is important to emphasize that the VRAS enabled the functional alignment strategy to be performed precisely and accurately, which would not be achievable if done conventionally.

## Conclusion

The VRAS demonstrated improved early postoperative advantages in the form of shorter surgical duration and LOS, as well as improved immediate postoperative ambulation distance. At 6 months, VRAS also showed significant improvements in SF-36 vitality, mental component summary, and a larger proportion of patients achieving SF-36 bodily pain MCID, with comparable pain, satisfaction, OKS, and KSS between groups. Our early results suggest that VRAS use may facilitate enhanced early recovery for patients and contribute to improved quality-of-life outcomes in the form of psychological benefits. Further studies with larger cohorts and longer follow-up duration are needed to determine its longer-term clinical value.

## Supplementary Information

Supplementary Material 1.

## Data Availability

The data that support the findings of this study are available on reasonable request from the corresponding author. The data are not publicly available due to privacy or ethical restrictions.

## Abbreviations

TKA: Total Knee Arthroplasty
rTKA: Robotic Total Knee Arthroplasty
cTKA: Conventional Total Knee Arthroplasty
OA: Osteoarthritis
VRAS: VELYS Robotic-assisted System
OKS: Oxford Knee Score
KSS: Knee Society Score
SF36: Short-Form 36
PCS: Physical Component Summary
MCS: Mental Component Summary
BMI: Body Mass Index
MCID: Minimal Clinically Important Difference
POD1: Post-operative day 1
VAS: Visual Analogue Scale
LOS: Length of Stay
RCT: Randomized Controlled Trial
