# Supplementary material for: VELYS robotic-assisted total knee replacement leads to improved mobility, reduction in hospitalisation, surgical duration, and better psychological outcomes: a propensity score matched analysis
Source: Arthroplasty. 2025 Nov 3;7:55. doi: 10.1186/s42836-025-00342-x (PMC12581327; doi:10.1186/s42836-025-00342-x)
Supplement: Supplementary file 1 — Supplementary Material 1. [file 42836_2025_342_MOESM1_ESM.docx]

**Supplementary Material**


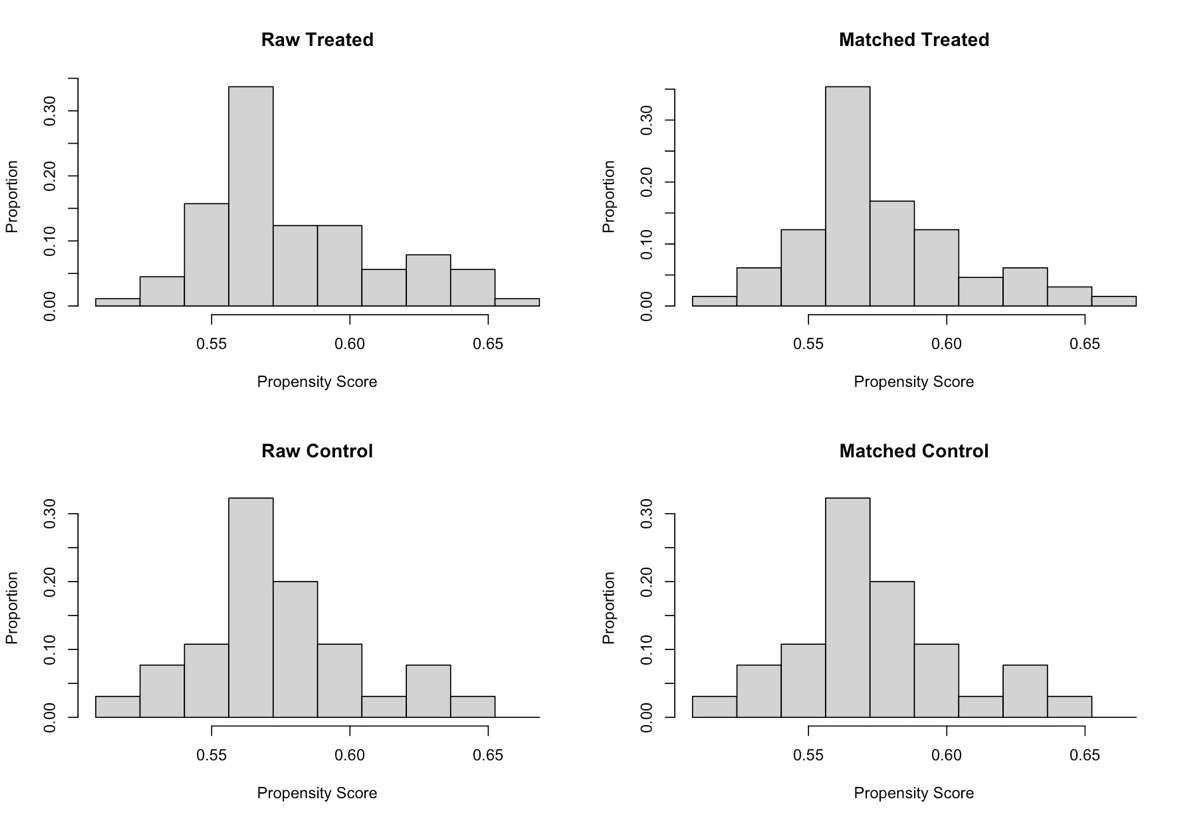


**Fig. S1.** Histogram of propensity scores for unmatched and matched cohorts


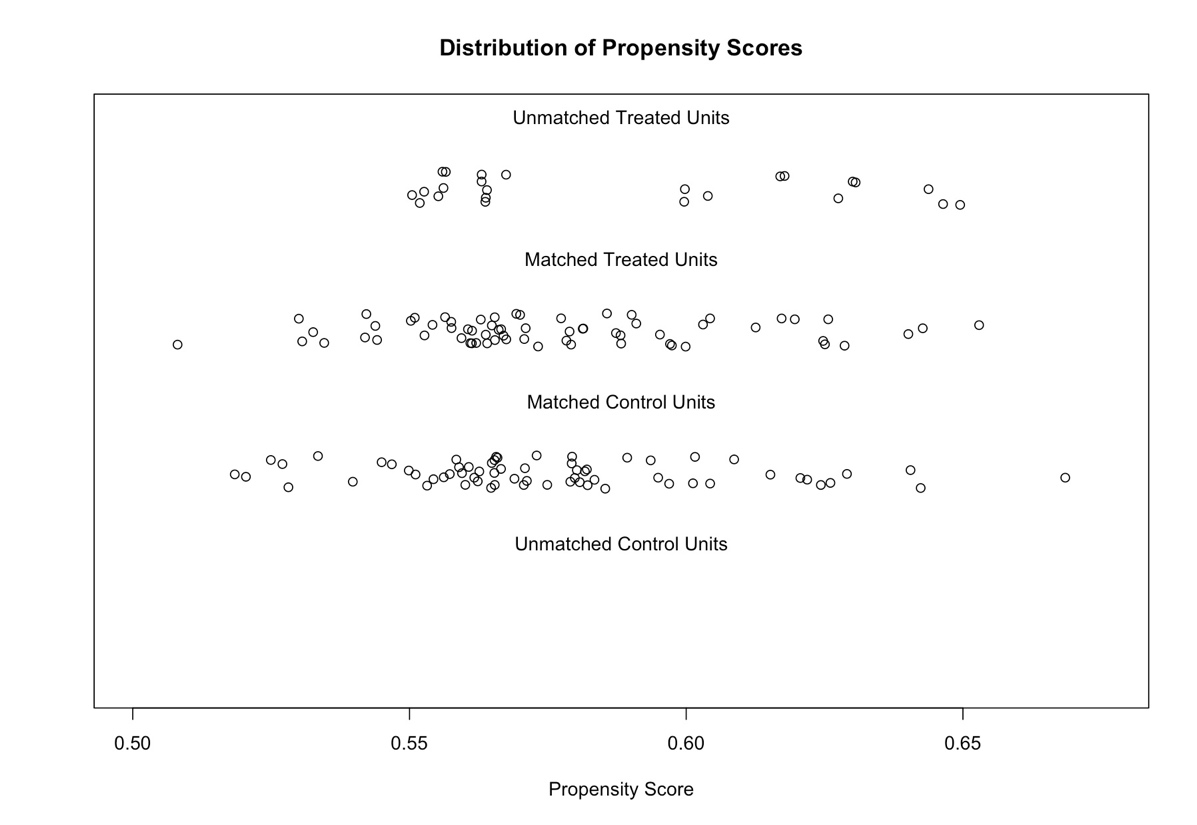


**Fig. S2.** Jitter plot of distribution propensity scores for unmatched and matched cohorts
